# Cascaded neural networks improving fish species prediction accuracy: the role of the biotic information

**DOI:** 10.1038/s41598-018-22761-4

**Published:** 2018-03-15

**Authors:** Simone Franceschini, Emanuele Gandola, Marco Martinoli, Lorenzo Tancioni, Michele Scardi

**Affiliations:** 1 Department of Biology, University of Rome Tor Vergata, via della Ricerca Scientifica 1, 00133 Rome, Italy; 2 Department of Mathematics, University of Rome Tor Vergata, via della Ricerca Scientifica 1, 00133 Rome, Italy

## Abstract

Species distribution is the result of complex interactions that involve environmental parameters as well as biotic factors. However, methodological approaches that consider the use of biotic variables during the prediction process are still largely lacking. Here, a cascaded Artificial Neural Networks (ANN) approach is proposed in order to increase the accuracy of fish species occurrence estimates and a case study for *Leucos aula* in NE Italy is presented as a demonstration case. Potentially useful biotic information (i.e. occurrence of other species) was selected by means of tetrachoric correlation analysis and on the basis of the improvements it allowed to obtain relative to models based on environmental variables only. The prediction accuracy of the *L*. *aula* model based on environmental variables only was improved by the addition of occurrence data for *A*. *arborella* and *S*. *erythrophthalmus*. While biotic information was needed to train the ANNs, the final cascaded ANN model was able to predict *L*. *aula* better than a conventional ANN using environmental variables only as inputs. Results highlighted that biotic information provided by occurrence estimates for non-target species whose distribution can be more easily and accurately modeled may play a very useful role, providing additional predictive variables to target species distribution models.

## Introduction

Developments in Machine Learning have resulted in an increasingly wider utilization of those methods in ecological and environmental modeling^1–3^ due to their ability to handle non-linear relationships and to provide accurate results in simulations. Especially, within a framework of global climate changing and increasing anthropic disturbance, the use of ML methods for assessing species occurrence and distribution has become a very important means to detect changes in environmental health^4,5^.

Artificial Neural Networks (ANNs) are increasingly used by scientists and policy makers in order to support water management strategies and environmental policies^6^. Particularly, predicting structure and diversity of fish assemblages under natural and anthropic disturbance and understanding which environmental factors are the most relevant to species distribution are fundamental aspects in conservation and management activities aimed at preserving freshwater ecosystems or restoring them to the optimal ecological status^7–9^.

Several studies used ANNs to elucidate the role of the main environmental variables involved in fish occurrence prediction^10–12^. Moreover, most of the studies were focused on the role of purely environmental factors in affecting species distribution and on the relationships between them^13–15^. The use of biotic information has only rarely been taken into account as a complementary source of input variables^16,17^.

It is well known in ecology that fish species distribution is affected both by environmental variables and biotic interactions such as interspecific competition or predation^18^. Therefore, biotic relationships affect likewise fish community structure, so defining a certain number of fish species combinations which may really exist. In fact, given a fish species assemblage containing *n* species, the theoretical number of combinations of fish species occurrences should be 2^*n*^, while ecological works have evidenced that they are far fewer^19^. While in most cases the reason for recurring fish assemblages may depend on species that share similar responses to environmental conditions, in some cases correlations in species distributions may highlight potential biotic interactions.

As combinations of fish species occurrences are not infinite and biotic interactions may affect fish species distribution, these relationships – even in case they are only the outcome of similar responses to environmental conditions - can be exploited in order to obtain better predictions of fish species occurrence. Several papers deal with methods aimed at investigating ecological interactions between fish species in freshwater ecosystems, e.g. using generalized linear models^20^ or mechanistic models, as proposed by Olden & Poff^21^. Here, we present an approach aimed at exploiting the information conveyed by potential ecological interactions between freshwater fish species, thus improving the accuracy of species distribution models. Highlighting potential ecological interactions between fish species may be considered a secondary valuable outcome of the proposed method, since they can be inferred on the basis of the gain in accuracy of an ANNs model when predicted occurrences of other species, which can be more easily modeled, are used as additional input variables. To demonstrate this approach, we tested several models based on the addition of occurrence data for other correlated species to a species distribution model aimed at *Leucos aula*, a thermophilic species characterized by an omnivorous diet (invertebrates, algae and aquatic macrophytes) that mainly occurs in water streams and lakes with slow current and plentiful benthic vegetation^22^. The selection of *L*. *aula* as the target species for demonstrating this new modeling approach was independent of conservation issues and only based on the good level of knowledge about its ecology and on the even balance between its presence and absence records, which made this species a good candidate for species distribution modeling.

Obviously, once the co-predictor species had been selected on the basis of the available field data, only their predicted occurrences were passed as inputs to the model aimed at predicting *L*. *aula*. As the output from one or more ANNs here becomes the input to another, this methodology can be referred to as cascaded neural networks and it has been already used in other ecological applications^23^. The main goal of this procedure was to select and exploit suitable biotic information, either causal or correlative, that is already available in any set of fish assemblage records that also includes the target species. Needless to say, information obtained from field work is only needed to train the cascaded ANNS, as estimated occurrences for co-predictor species are obtained from dedicated ANNs and passed at run time to the ANN aimed at predicting the occurrence of *L*. *aula*.

## Materials and Methods

### Data collection and sampling sites

**Figure 1:**
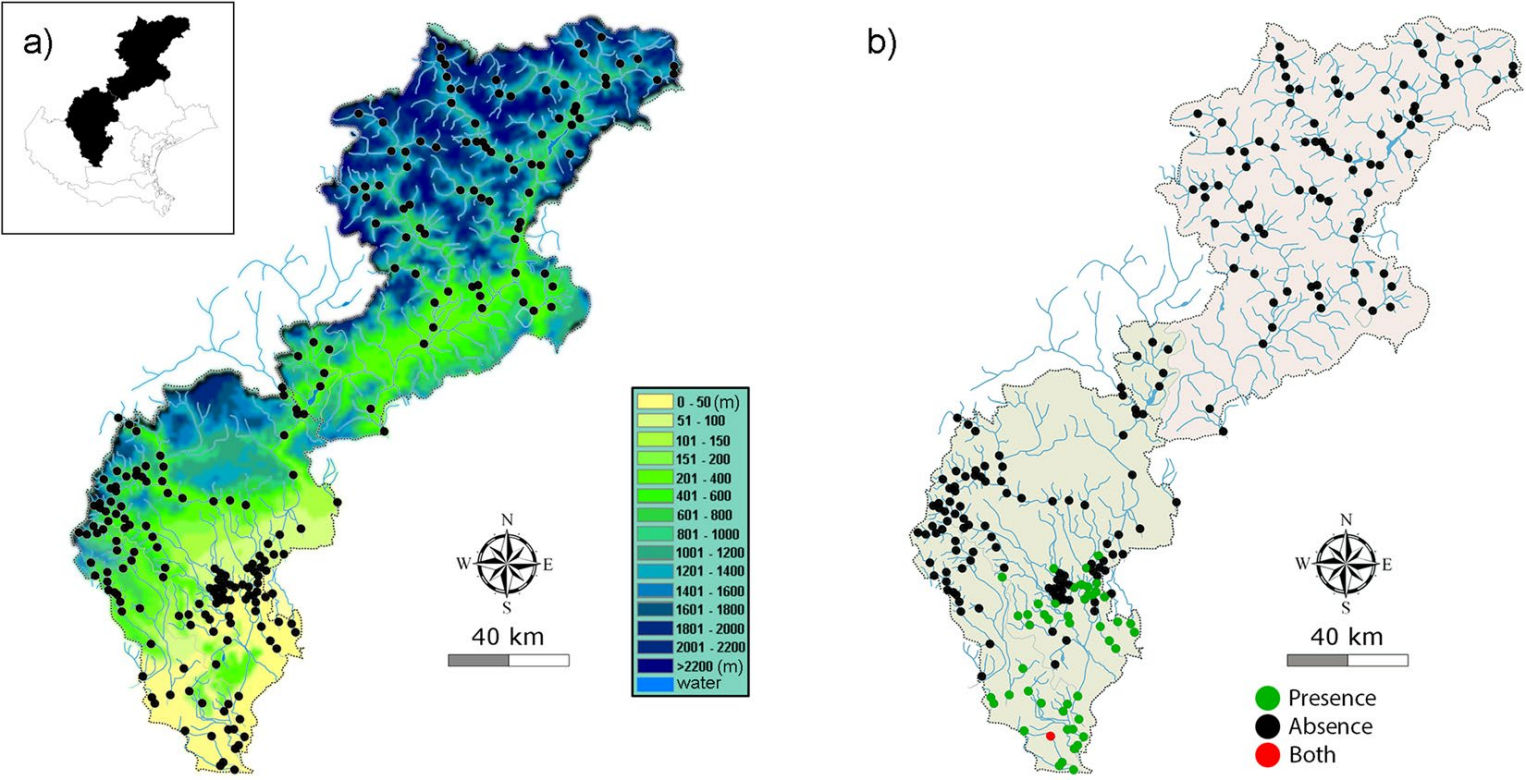
Sampling sites. Veneto river basins, NE of Italy. (**a**) Elevation map of the river basins. BLACK dots mark the position of the sample sites. (**b**) *L*. *aula* occurrence in the river basins. GREEN dots mark presence, RED both presence and absence (same site, different times), BLACK absence. Images were obtained by using QGIS software^51^ (http://grass.osgeo.org). Original image was generated by Michele Scardi and then processed by Emanuele Gandola using Adobe Photoshop cs6 (Version 13.0).

Data have been obtained from 264 samples that have been collected from 1991 to 1995 and published in report about the fish fauna of the Veneto region (north-eastern Italy, Fig. 1) by Zanetti *et al*.^24^ and Salviati *et al*.^25^. Seasonal sampling activities in the same sites have been stored in the database as different records to represent the local inter-annual variability of both environmental variables and fish fauna. Fish assemblage composition was recorded as binary presence/absence data for 34 fish species (Table 1). The values of 20 environmental variables (Table 2) were also recorded during fish sampling. Most of these variables had been already considered in previous studies^26–29^.

**Table 1:** List of the fish species in the Veneto data set.

N	Scientific name	English name
1	*Leucos aula (Bonaparte, 1841)*	(Triotto)
2	*Padogobius bonelli (Bonaparte, 1846)*	Padanian Goby
3	*Scardinius erythrophthalmus (Linnaeus, 1758)*	Rudd
4	*Esox lucius (Linnaeus, 1758)*	European Pike
5	*Squalius cephalus (Linnaeus, 1758)*	Chub
6	*Alburnus arborella (Bonaparte, 1841)*	Bleak
7	*Cottus gobio (Linnaeus, 1758)*	Bullhead
8	*Tinca tinca (Linnaeus, 1758)*	Tench
9	*Cobitis taenia (Linnaeus, 1758)*	Spined loach
10	*Phoxinus phoxinus (Linnaeus, 1758)*	Minnow
11	*Anguilla anguilla (Linnaeus, 1758)*	European Eel
12	*Knipowitschia punctatissima (Canestrini, 1864)*	Italian Spring Goby
13	*Salmo marmoratus (Cuvier, 1817)*	Marble Trout
14	*Sabanejewia larvata (DeFilippi, 1859)*	Italian Loach
15	*Ameiurus melas (Rafinesque, 1820)*	Black Bullhead
16	*Lepomis gibbosus (Linnaeus, 1758)*	Pumpkinseed
17	*Barbus plebejus (Bonaparte, 1839)*	Italian Barbel
18	*Protochondrostoma genei (Bonaparte, 1839)*	South Europe Nase
19	*Gasterosteus aculeatus (Linnaeus, 1758)*	Three-spined Stickleback
20	*Carassius carassius (Linnaeus, 1758)*	Crucian Carp
21	*Gobio gobio (Linnaeus, 1758)*	Gudgeon
22	*Telestes souffia (Risso, 1827)*	Blageon
23	*Thymallus thymallus (Linnaeus, 1758)*	Grayling
24	Lampetra planeri (Bloch, 1784)*	Po Brook Lamprey
25	Gambusia holbrooki (Girard, 1859)*	Eastern mosquitofish
26	Barbus meridionalis (Risso, 1827)*	Mediterreanean Barbel
27	Micropterus salmoides (Lacepède, 1802)*	Large-Mouthed Bass
28	Perca fluviatilis (Linnaeus, 1758)*	Perch
29	Abramis brama (Linnaeus, 1758)*	Common Bream
30	Cyprinus carpio (Linnaeus, 1758)*	Common Carp
31	Salvelinus fontinalis (Mitchill, 1814)*	Brook Char
32	Salmo trutta (Linnaeus, 1758)**	Sea Trout
33	Oncorhynchus mykiss (Walbaum 1792)**	Rainbow Trout
34	*Salmo(trutta) hybr*. trutta/marmoratus**	Sea Trout-Marble Trout hybrid

Taxa on white background were used in the models while grey background highlights the excluded species. Scientific names were revised according to the current classification. The Italian name is shown in brackets for the only species with no English name. *Taxa excluded since their presence records were <10. **Taxa excluded regardless of their rarity because their occurrence depends on stocking programmes.

**Table 2:** Environmental descriptors used as input (i.e. predictive) variables.

Variable	Min	Max	Mean	Median
Elevation (m)	13.00	1785.00	400.92	260.00
Mean depth (m)	0.01	1.46	0.45	0.40
Runs (area, %)	0.00	100.00	55.14	55.00
Pools (area, %)	0.00	90.00	14.79	5.56
Riffles (area, %)	0.00	100.00	30.00	22.03
Mean width (m)	1.00	80.00	9.32	6.00
Boulders (area, %)	0.00	100.00	17.01	10.00
Rocks and pebbles (area, %)	0.00	100.00	29.97	30.00
Gravel (area, %)	0.00	96.00	21.48	15.00
Sand (area, %)	0.00	80.00	7.99	4.50
Silt and clay (area, %)	0.00	100.00	23.44	0.00
Stream velocity (score, 0–5)	0.00	5.00	0.00	0.00
Vegetation cover (area, %)	0.00	100.00	10.85	0.00
Shade (%)	0.00	100.00	37.86	40.00
Anthropic disturbance (score, 0–4)	0.00	4.00	1.45	1.60
pH	5.63	9.33	7.75	7.76
Conductivity (µS cm^−1^)	11.00	1851.00	406.63	390.00
Gradient (%)	0.02	41.60	4.38	1.38
Catchment area (km2)	0.34	3274.01	169.82	19.71
Distance from source (km)	0.33	119.27	16.79	7.14

Elevation data were obtained by cartographic or *in situ* GPS measurements. Mean depth was measured by a graduated pole. All the percentages about the mesohabitat characteristics (runs, pools, riffles) and the particle size of sediment (boulders, rocks and pebbles, gravel, sand, silt and clay) were visually estimated by the operator. Stream velocity was measured by hydrometric paddle-wheels and it was converted to semi-quantitative values (0 = still waters; 1 = 5–6 cm/s; 2 = 7–30 cm/s; 3 = 35–50 cm/s; 4 = 55–100 cm/s; 5 =  >100 cm/s). Vegetation cover (i.e. the percentage of the stream channel covered by aquatic macrophytes) as well as shade were visually estimated by the operator. The anthropic disturbance takes into account hydromorphological alterations of the rivers due to increasing anthropic impacts (channel shape, urbanization, etc.) and it was visually estimated by the operator. The conductivity and the pH values were evaluated by the use of handheld instruments.

Although geographical coordinates can be regarded as proxies for other variables that are not explicitly included in the data set in any kind of empirical model, included those based on Machine Learning techniques, they were not used to avoid biases related to spatial autocorrelation.

Fish were sampled using a standard electro-fish shoulder-bag (4KW, 0.3–6 Ampere, 150–600 Volt) and all available habitats were sampled along a stream channel 40–70 m long (the transect length was about 10 times the width of the wetted channel).

Fish sampling met all relevant ethical safeguards and all captured fishes were anesthetized with 0.035% MS 222 solution (Tricaine 92 Methanesulfonate) and photographed before release.

### Data set processing

To reduce biases in model development, eight taxa with low occurrence were excluded (<10 samples, marked with an asterisk in Table 1). In fact, difficulties of ANNs in identifying distribution patterns of rare species could easily led to incorrect predictions^30^.

Moreover *Oncorhynchus mykiss*, *Salmo trutta* and *Salmo (trutta)* hybr. *trutta/marmoratus* were excluded regardless of their rarity since their occurrence does not depend on environmental conditions alone. Indeed both *O*. *mykiss* and *S*. *trutta* distribution is strictly related to the artificial release of reared juveniles, while distribution of *Salmo (trutta)* hybr. *trutta/marmoratus* is partly correlated to the occurrence of the two parental species.

Fish fauna occurrence has been coded by binary values (0–1), i.e. absence or presence respectively, while quantitative or semi-quantitative environmental variables were normalized in a [0, 1] interval.

### Species correlation

The tetrachoric correlation coefficient, which is analogous to the Pearson correlation coefficient, but aimed at binary data, was computed between *L*. *aula* and other fish species in R^31^ with the package psych^32^.

### Artificial Neural Network models

#### Models architecture

In this study, several models based on ANNs were developed and optimized to predict *L*. *aula* occurrence. ANNs were trained and tested by using the nnet^33^ function of R, considering three layered feed-forward neural networks with bias. The performance of different networks (with 1 to 15 hidden neurons) were compared in order to choose the best network configuration. A sigmoid transfer function was used both for hidden and output layer, so enabling the network to learn non-linear relationships between input and output vectors. The ability to easily handle non-linear relationships^34^ is a very useful feature of ANNs, especially when dealing with highly complex data sets.

#### Model development

**Figure 2:**
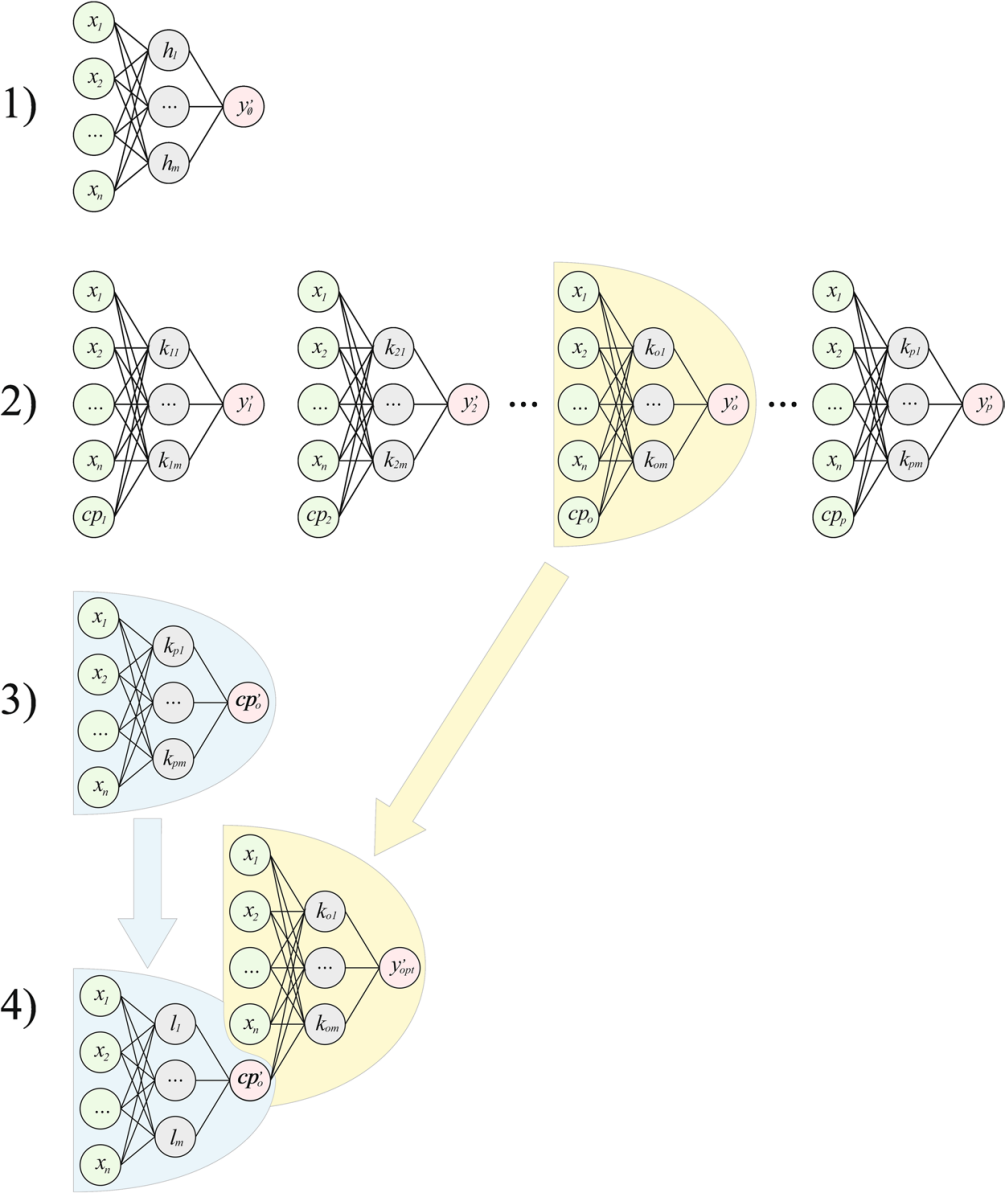
Model development. The general procedure for training a cascaded ANN model involves four steps: 1) an ANN aimed at predicting the target species (*y)* is trained with *n* environmental variables (*x*) only as inputs and its output is analyzed to establish the baseline performance level; 2) *p* ANNs are trained to predict the same target species, using the same *n* environmental variables and an additional input based on the occurrence records for each one of the *p* remaining species, one at the time, thus identifying the species whose addition as co-predictor provides the largest performance improvement in relative to step 1; 3) an ANN aimed at assessing the expected occurrence of the most effective co-predictor species, according to step 2, is trained using as inputs the *n* environmental variables only; 4) a cascaded ANN model aimed at predicting the target species is obtained by combining the best ANN from step 2 and the one from step 3. The cascaded ANN model needs observed data for the environmental variables only, while biotic information is provided through sub-model predictions and therefore is not needed to run the model. Green ANN input nodes require field data, while pink ANN nodes provide or require predicted values. Only a single co-predictor species is shown, but a very similar procedure can be applied if more co-predictor species are used.

The ANN model development was based on the following general procedure (Fig. 2):

1. an ANN aimed at predicting the target species occurrence is trained with *n* environmental variables as inputs and its output is analyzed to establish the baseline performance level;
2. *p* ANNs predicting the target species are trained with the same *n* environmental variables and with an additional input based on occurrence records for each one of the *p* remaining species, one at the time (this step is aimed at finding out the potential contribution of known biotic information, i.e. species occurrence, to the target species predictions, thus identifying the species whose addition as co-predictor provided the largest improvements relative to step 1);
3. an ANN aimed at assessing the expected occurrence of the most effective co-predictor species, according to step 2, is trained using as inputs the *n* environmental variables only;
4. a cascaded ANN model aimed at predicting the target species occurrence is obtained by combining the best ANN from step 2 and the one from step 3.

In case more than a single co-predictor species may play a useful role, the procedure can be modified in order to exploit the biotic information they contribute to the model. This requires training one more ANN at step 2, using all the *k* co-predictor species as *k* additional inputs to the same ANN, and *k* ANNs at step 3, one for each co-predictor species. The final cascaded ANN model will be comprised of the ANN with *k* co-predictors species as additional inputs and of *k* ANNs aimed at predicting the occurrence of each co-predictor species on the basis of environmental inputs only. The latter ANNs pass their output to the input layer of the former, thus allowing the resulting model to predict the target species occurrence on the basis of environmental input variables only.

The cascaded ANNs approach will be here demonstrated using two co-predictor species.

#### Post-processing of model outputs

Model optimization was performed using the Receiver Operating Characteristic (ROC) curves^35,36^. Ideally, the neutral cut-off to discriminate presence/absence predictions, i.e. to binarize output from ANNs, should be 0.5. However, unbalanced numbers of presence and absence cases in training data often lead to output values whose distribution can be better binarized by a different threshold value, thus minimizing false positive (FP) and false negative (FN) results^37^.

ROC curve analysis was performed to define the best threshold value for each model, taking into account the test set. The evaluation of the ROC curves was performed using the R package pROC^38^.

#### Model validation

Models performance was evaluated using five-fold Cross-validation (CV)^39^. A confusion matrix was computed for each model to show true positive (TP), false positive (FP), false negative (FN) and true negative (TN) predicted cases.

The prediction error of the models was assessed by the Cohen’s kappa (*K*) coefficient^40^, which measures the deviation of model predictions from those of a random process:

1 $$k=\frac{(TP+TN)-[((TP+FN)(TP+FP)+(FP+TN)(FN+TN))/n]}{n-[((TP+FN)(TP+FP)+(FP+TN)(FN+TN))/n]}$$

While the deviation from random predictions may be formally tested, Kappa values can be also interpreted heuristically using the scale proposed by Landis and Koch^41^.

#### Sensitivity analysis and perturbation method

In order to assess the contribution of each input variable to the ANNs estimation process three methods were chosen:

- A sensitivity analysis was carried out according to the “profile” method proposed by Lek^10,42^. The scale (i.e. the number of intervals in which each variable is divided) was set to 50; while all other variables were set at their minimum values, first quartile, median, third quartile and maximum.
- The “perturbation” method was applied following the approach proposed by Scardi and Harding^43^. White noise in the [−0.3, 0.3] range was added to each input variable while keeping the values for all the others untouched.
- The “weights” method, proposed by Olden *et al*.^13^, was also applied. This method calculates the importance of each variable as the product of the raw connection weights between each input-output neuron and sums the product across all hidden neurons. The sign of the contribution shows if increasing values of the predictive variable are positively or negatively correlated to the expected probability of species presence.

Through these methods we wanted to highlight variables that play a major role in the prediction process. This result can be useful to infer potential causal relationships or to select variables that are good candidates for developing a simpler model^44^. While applying these methodologies to reduce the number of input variables is a typical goal with ANNs modeling^45^, to demonstrate the cascaded neural network approach we decided to keep the entire set of variables, which includes those that are more commonly included in freshwater fish community modeling^10,27–29^. In fact, the *a posteriori* selection of an effective subset of predictors was certainly possible, but it was not relevant to the goal of this study, which is to show that predictions about the occurrence of a species can be improved by using predictions about other (easier to predict) species. Therefore, in order to demonstrate this modeling strategy, keeping the same set of input variables for each model was much more convenient and allowed to obtain fully comparable results from different options. Obviously, in case the curse of dimensionality^46^ impaired the training procedure, which was not the case with our data, then selecting a subset of input variables could have been necessary.

### Data availability

All data generated or analysed during this study are included in these published articles: Zanetti *et al*.^24^ and Salviati *et al*.^25^.

## Results

### L. aula prediction

The first model generated for *L*. *aula* prediction was built with environmental variables only as inputs to the ANN. ROC curve analysis showed that the optimal cut-off value for binarizing the ANN output was 0.548. The model prediction on test set data was improved from a *K* value of 0.582 to 0.627 (confidence interval: 0.410–0.805) using the ROC curve cut-off value. The confusion matrix shown in Table 3 is the one associated to the median K value obtained by 5-fold cross-validation.

**Table 3:** Confusion matrix obtained by *L*. *aula* prediction on testing set.

		Observed	
		Absence (0)	Presence (1)
Predicted	Absence (0)	35	3
	Presence (1)	5	10

**Figure 3:**
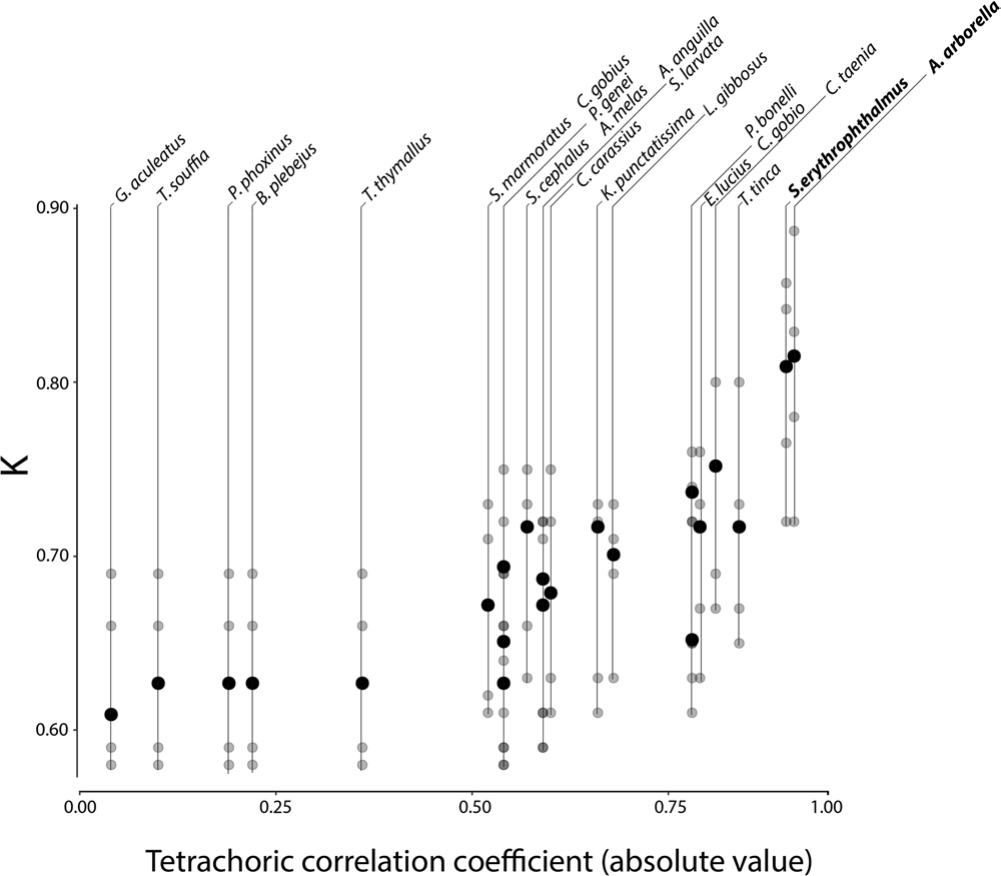
Results obtained by addition of correlated species. *K* values of models obtained by the addition of an additional co-predictor species relative to their tetrachoric correlation coefficient with *L*. *aula*. Species whose addition significantly increased the *K* value, i.e. above the upper limit of the confidence interval of the model based on environmental variables only, i.e. [0.410,0.805], are marked in bold. Grey dots represent results from 5each fold in the 5-fold cross-validation. Image was obtained by using R software^31^.

The ranking of *K* values obtained from models trained by adding occurrence information for an additional species to the ANN inputs are shown in Fig. 3. No improvements in model performance were observed when species whose occurrence was loosely correlated to *L*. *aula* records were added as co-predictors. In fact, addition of species with null to weak tetrachoric correlation to *L*. *aula* (i.e. with *r* ranging between −0.04 and 0.54) did not provide better *K* values than the original model with no biotic co-predictors. By contrast, using species whose correlation to *L*. *aula* (in absolute value) was higher than 0.54 as additional ANN inputs allowed to improve model performance, although the resulting *K* values were not strictly proportional to the value of the tetrachoric correlation coefficient (Fig. 3). The largest increase in model accuracy was obtained by the addition of *A*. *arborella* and *S*. *erythrophthalmus* occurrence information to the model, reaching *K* values of 0.815 and 0.809 respectively, exceeding in both cases the upper limit of the *K* confidence interval obtained for the first model *K* (0.410–0.805). Confusion matrixes derived by the addition of each one of those species are shown in Tables 4, 5.

**Table 4:** Confusion matrix obtained by the addition of *A*. *arborella* observed occurrence as input variable.

		Observed	
		Absence (0)	Presence (1)
Predicted	Absence (0)	36	0
	Presence (1)	4	13

**Table 5:** Confusion matrix obtained by the addition of *S*. *erythrophthalmus* observed occurrence as input variable.

		Observed	
		Absence (0)	Presence (1)
Predicted	Absence (0)	37	1
	Presence (1)	3	12

### L. aula prediction via cascaded ANNs

Expected probabilities of occurrence of *A*. *arborella* and *S*. *erythrophthalmus* were then used to improve the learning process of the model for *L*. *aula* via the cascaded ANNs approach.

The model for *A*. *arborella* occurrence prediction showed a *K* value of 0.708 relative to the test set, while the *K* value for *S*. *erythrophthalmus* model was 0.659. Both *K* values were obtained from the optimized model using the binarization cut-off values from ROC curves, i.e. 0.603 and 0.571, for *A*. *arborella* and *S*. *erythrophthalmus* respectively.

*L*. *aula* prediction models were improved by using the predicted occurrence probabilities of the two species as co-predictors, i.e. as new input variables in secondary ANNs. Results (Tables 6,7) showed *K* values of 0.729 and 0.697 for the *L*. *aula* model using predicted *A*. *arborella* and *S*. *erythrophthalmus* presence probabilities as co-predictors.

**Table 6:** Confusion matrix obtained by the addition of *A*. *arborella* predicted occurrence as input variable.

		Observed	
		Absence (0)	Presence (1)
Predicted	Absence (0)	35	1
	Presence (1)	5	12

**Table 7:** Confusion matrix obtained by the addition of *S*. *erythrophthalmus* predicted occurrence as input variable.

		Observed	
		Absence (0)	Presence (1)
Predicted	Absence (0)	36	2
	Presence (1)	4	11

Finally, another model was trained using both species presence probabilities as co-predictors, thus obtaining a *K* value of 0.765 (Table 8). The “profile”, “perturbation” and “weights” sensitivity analyses were performed on this model.

**Table 8:** Confusion matrix obtained by the addition of both species predicted occurrences as input variables.

		Observed	
		Absence (0)	Presence (1)
Predicted	Absence (0)	36	1
	Presence (1)	4	12

### Variables importance

**Figure 4:**
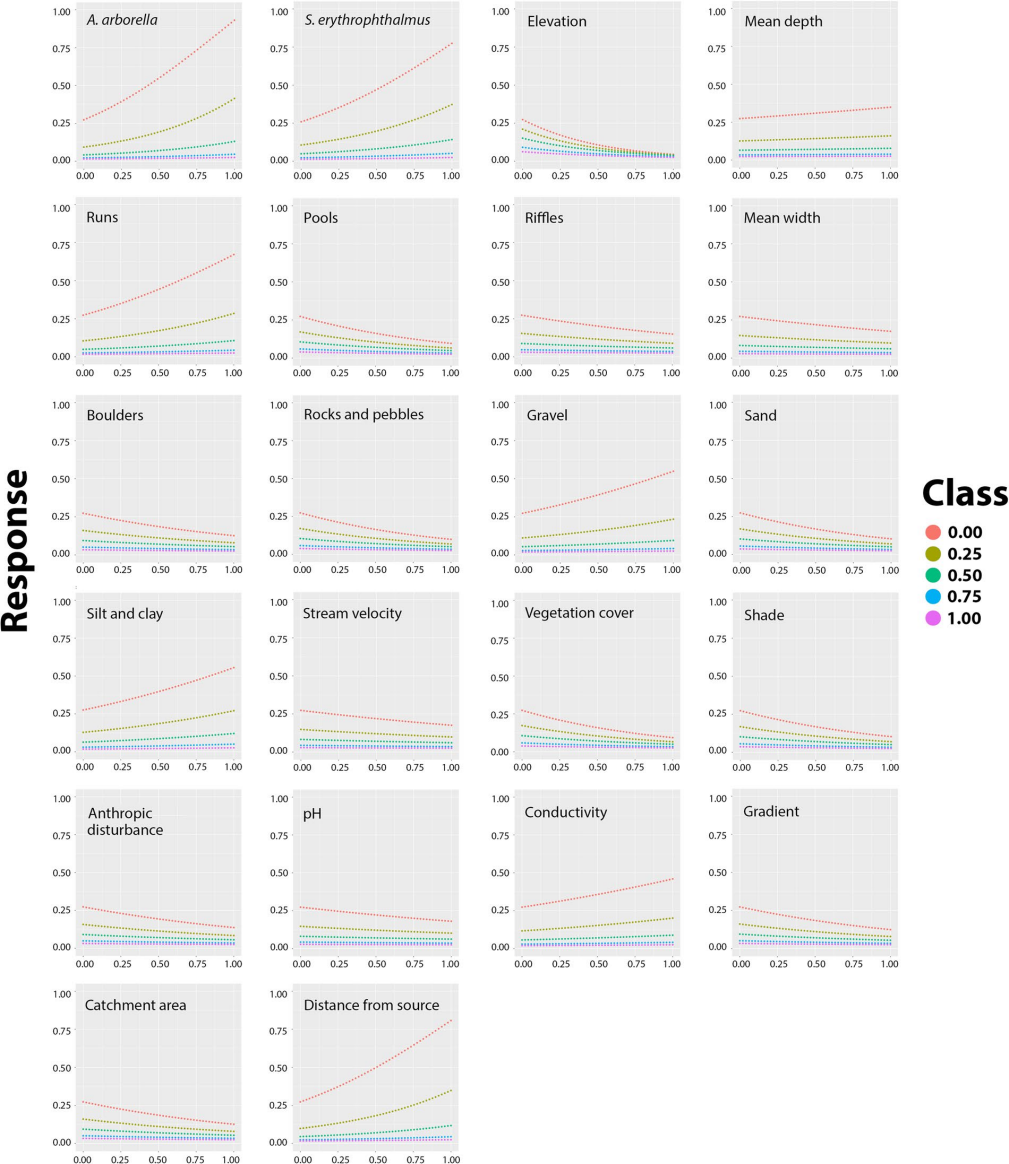
Lek’s “profile” method for sensitivity analysis. The occurrence expected probability of *L. aula* (“Response”) at increasing values of each input variable, keeping all the others normalized inputs at five fixed levels ranging from 0 to 1 with a 0.25 step, thus generating five response curves. Images were obtained by using R software.

The results of the “profile” method are shown in Fig. 4. Graphs illustrate the responses of the ANN to variations of each input variable. Results showed that modeled occurrence probabilities for both co-predictor species positively contributed to the estimation of *L*. *aula* occurrence.

**Figure 5:**
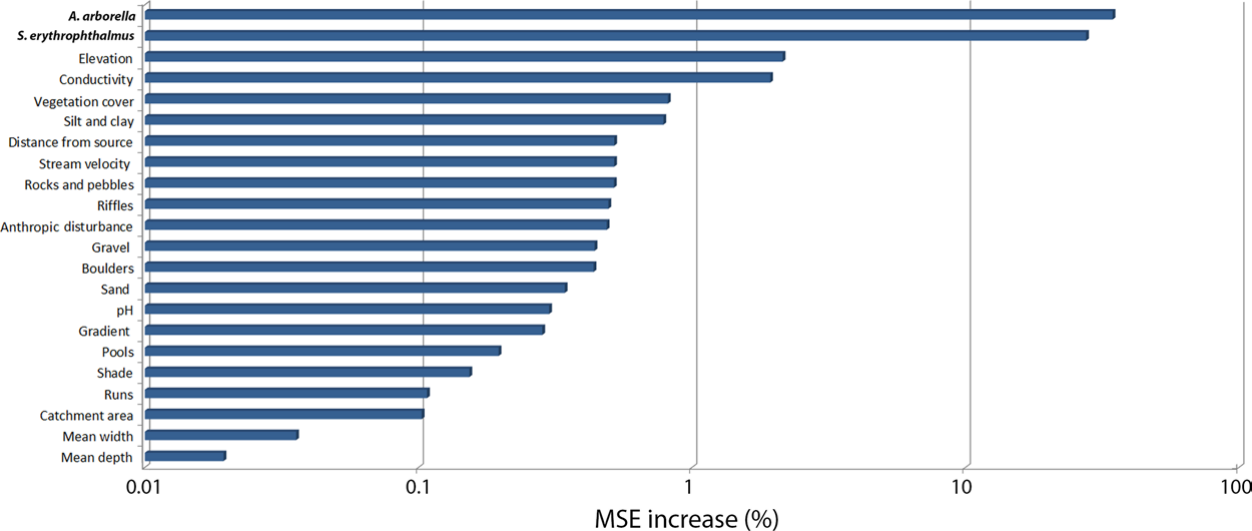
“Perturbation” method for sensitivity analysis. Percent increase in mean square error of the ANN output obtained by perturbation of the test set data patterns. White noise in the [−0.3, 0.3] range was added to each value of each input variable, while keeping all the other inputs at their original values. Image was obtained by using R software.

In Fig. 5 the results obtained with the “perturbation” method are shown. For each variable, increasing white noise additions caused increasing mean square error values in the output. While the expected probabilities of occurrence were only the output of an ANN, i.e. they were not real values, they proved to be the most influential predictive variables in the estimation process for *L*. *aula*.

**Figure 6:**
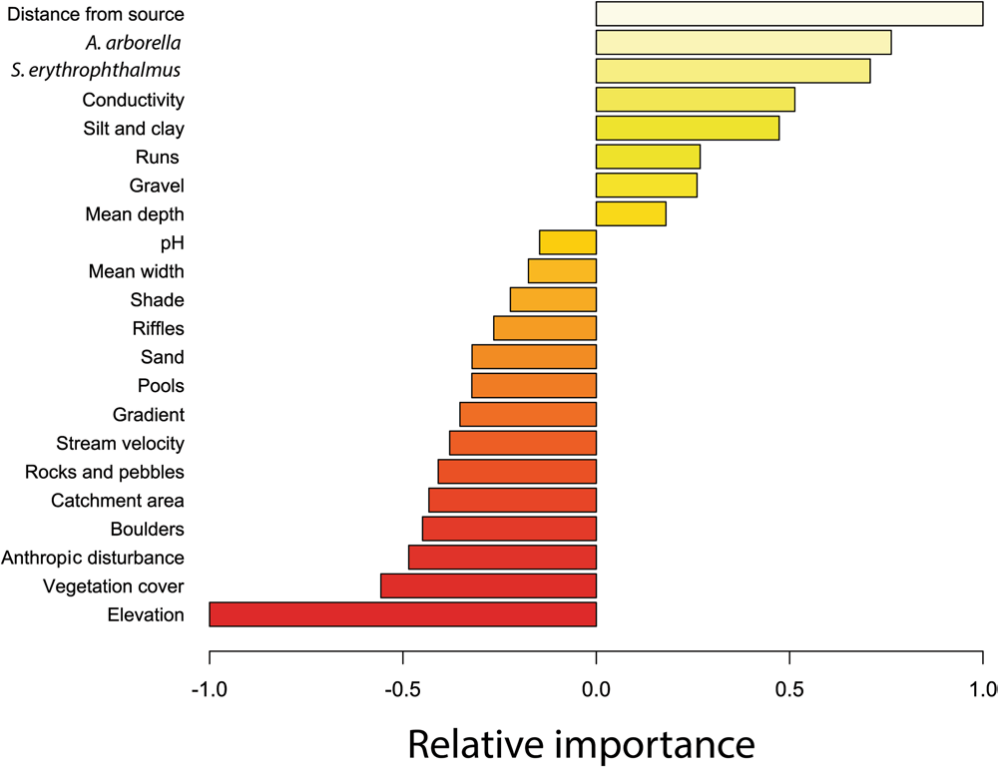
“Weights” method for variable imprtance. Relative importance of input variables is assessed on the basis of ANN weights. Negative contributions of input variables imply negative correlation between predictive variables and *L*. *aula* occurrence (e.g. probability of presence is expected to be low at high elevation sites). Image was obtained by using R software.

The relative contributions of the input variables to the prediction of *L*. *aula* according to the “weights” method are shown in Fig. 6. The predictive variables with the highest positive relative contributions were distance from source, *A*. *arborella*, *S*. *erythrophthalmus* and conductivity. Elevation and anthropic disturbance showed a high contribution on the occurrence estimation of *L*. *aula* from a negative point of view (i.e. for increasing values of these predictive variables a low probability of *L*. *aula* presence was expected).

## Discussion

Our results showed how an ANN model aimed at predicting *L*. *aula* occurrence achieved different levels of accuracy depending on the addition of correlated species as biotic co-predictors. Taxa that show a high positive correlation with *L*. *aula* share its main ecological features, e.g. tolerance to low oxygen and habitat preference for slow current^22^, and therefore respond in a similar way to environmental conditions. However, some of them were easier to predict than *L*. *aula*, while their distribution could be regarded as a proxy for complex environmental features that in turn may implicitly play a role in driving the distribution of *L*. *aula*. Therefore, they can be useful as co-predictors, even when their occurrence is unknown, because their modeled distribution is reliable enough to be used instead of field data.

ANN models which included as co-predictors observed data about the occurrence of the most correlated species to *L*. *aula*, i.e. *A*. *arborella* and *S*. *erythrophthalmus*, (*r* = 0.91 and *r* = 0.90 respectively), showed the highest accuracy. *K* was equal to 0.815 for *A*. *arborella* and 0.809 for *S*. *erythrophthalmus*, in both cases exceeding the upper limit of *K* confidence interval of the *L*. *aula* model based on environmental variables only (Fig. 3). Using the occurrence of the two most correlated species as additional input information the model performance improved from “good” to “very good” according to the scale of *K* of agreement by Landis and Koch^41^. In this case, model improvements depended on the biotic information conveyed by strongly correlated species, which indirectly suggested where environmental conditions were potentially suited for *L*. *aula* presence. Indeed the presence of *A*. *arborella* and *S*. *erythrophthalmus* could be regarded as an indicator of the river traits where *L*. *aula* is more likely to occur. On the other hand, the addition of species like *S*. *cephalus* and *C*. *gobio* as co-predictors also improved the accuracy of the model regardless the strength of their correlation to *L*. *aula* (Fig. 3). This suggests that in some cases the improvement in model performance is not due to co-occurrence factors, while higher order relationships may play a role in affecting the learning process of the model. In fact, complex ecological relationships between species can be easily exploited thanks to the ability of ANNs to handle non-linear relationships between input variables^42^. This could be an important issue from an ecological perspective, because ANNs models could point out relationships between fish species that in some instances are independent of co-occurrence factors.

However, in most cases the performance of ANN models was not increased through the use of weakly correlated species. In fact, species with tetrachoric correlation between −0.36 and 0.54 provided no improvement, with the exception of *P*. *genei*. These species indeed may seem to share part of the distribution of *L*. *aula*, but they are usually found in a transition zone characterized by fast water current where *L*. *aula* is absent^22^. Finally, weakly correlated species (e.g. *G*. *aculeatus*) only added noise that could induce a decrease in prediction accuracy. In this case model accuracy was even lower than using only the standard set of environmental variables as inputs (*K* = 0.609). In fact, *G*. *aculeatus* presence is strictly correlated to spring-fed pools^47^ and therefore its co-occurrence with *L*. *aula* is completely random.

At the same time, the introduction of strongly negatively correlated species made it possible to improve model performance as much as with the positively correlated ones. In this case, improvement in model predictions was obtained by exclusion factors, as the presence of *S*. *marmoratus* and *C*. *gobio* suggested different features of the stream ecosystem, becoming a good predictor for *L*. *aula* absence.

Using predicted probabilities of occurrence of either *A*. *arborella* or *S*. *erythrophthalmus* as additional inputs improved estimates of *L*. *aula* presence (*K* = 0.729 and *K* = 0.697 respectively). While these *K* values were lower than those obtained by using observed data for those co-predictors (see Fig. 3), the addition of the estimated probabilities of occurrence of one of the co-predictor species improved the *L*. *aula* ANN model based on environmental variables only. This was a logical outcome, as their predicted probability of occurrence, although quite accurate, could not entirely match the real species distribution and therefore their effectiveness as co-predictors was partly reduced.

The addition of predicted probabilities of occurrence for both *A*. *arborella* and *S*. *erythrophthalmus* provided a further improvement in the *L*. *aula* model accuracy (K = 0.765). This result proved that combinations of two or more co-predictor species may allow to further improve the accuracy of cascaded ANN models, which obviously can be used by passing them only data about environmental variables.

Lek’s “profiles” in Fig. 4 pointed out that both co-predictor species significantly contributed to the estimation of *L*. *aula* occurrence by the ANN model. In particular, as the occurrence probabilities for the two species increased, an increase in the probability of *L*. *aula* occurrence was also expected.

These results provided a useful insight into the cascaded ANNs. In fact, as the *A*. *arborella* and *S*. *erythrophthalmus* occurrence probabilities are the output of independent ANNs, their values are the results of specific environmental patterns which can be indirectly passed to the second ANN^21^, which is aimed at predicting *L*. *aula*. For this reason, their predicted probabilities of occurrence enhance the presence or absence estimation for *L*. *aula* at any given site.

It is therefore not surprising that the results obtained from “perturbation” sensitivity analysis (Fig. 5) proved that the ANN model was more sensitive to variations in co-predictor species occurrence probabilities than to any environmental variable. In fact, as soon as the biotic information was added to the ANNs as co-predictor variables, most environmental variables seemed to play a less important role in estimating *L*. *aula* occurrence.

“Weights” method (Fig. 6) showed that presence probabilities for *A*. *arborella* and *S*. *erythrophthalmus* are positively correlated with the *L*. *aula* presence probability. This result confirmed that high probabilities of presence of both species provide valuable information about the environmental conditions at any given site where *L*. *aula* is to be predicted. From an ecological point of view, these results explain how the occurrence of *A*. *arborella* and *S*. *erythrophthalmus* could convey ecosystem information that could not be inferred from any single environmental variable. The information added by the predicted probabilities of occurrence of the two species became an important input signal to the ANN because their potential occurrence reinforces the effect of suitable environmental conditions for *L*. *aula* presence. The cascaded ANNs approach significantly improved *L*. *aula* prediction by 22% of the K value (K = 0.765 against K = 0.627 for the first model). Other approaches considered the biotic information as additional input variable in predictive models, in particular ANN^16,23^. Despite the good results that have been obtained by similar modeling procedures, biotic information has almost ever been used in the form of observed values. The use of predictions from other independent ANNs as additional input signals allowed to apply the *L*. *aula* ANN model even at sites where no biotic information was directly available, but where it could be estimated on the basis of environmental variables.

## Conclusions

Several authors explained how complex dynamics occur in predicting species distribution, since it is the result of complex relationships involving physical, chemical and biotic factors. Identifying biotic interactions between fish species can be very difficult, since indirect or high order relationships can be present. The main goal of this study was to show that better prediction of a species (here *L*. *aula* was used to demonstrate the approach) can be obtained by adding predicted probabilities of occurrence of correlated species as additional inputs. Moreover, our results suggest that potential interactions between species can be highlighted by analyzing model performances. Indeed, changes in ANN accuracy induced by additional co-predictor species suggests different levels of potential interaction between *L*. *aula* and other taxa, which in some cases are independent of co-occurrence factors, since model prediction improvements occur even with intermediate correlations between species, as in the case of *S*. *cephalus*. From this perspective, improvements in an ANN model may be regarded as a clue for the existence of ecological interactions between fish species, which obviously have to be further analyzed and eventually confirmed by more specific approaches.

The methodological framework here proposed provided higher predictive accuracy than conventional ANN models on the basis of the selection of correlated species as co-predictors. The most relevant co-predictor species were chosen on the basis of significant improvements in K values. This allowed to apply a selection criteria which provided only useful input information to the cascaded ANN without overly increasing its complexity. Using expected probabilities of fish occurrence as additional input variables implies that estimated biotic information can be added to the learning process of ANN models rather than observed biotic information, which would severely limit the practical value of the models.

As Scardi *et al*.^19^ also evidenced, the use of ANNs or related models in order to obtain more accurate prediction of fish species distribution cannot be really effective without the incorporation of approaches with an ecological perspective. In fact, conventional modeling methods may be unable to explain the complexity of the biotic systems and their interactions^18^. The direct or indirect relationships between species are relevant factors which significantly affect the fish assemblage composition, so the incorporation of biotic knowledge shall be considered as a focal point in species distribution modeling^48^. Of course there is a clear evidence that biotic interactions between species can change among different ecosystems^49,50^. Moreover, different selection criteria can be applied in order to choose which species may be relevant to the prediction process in ANNs. On this basis, several approaches may be considered in future in order to improve cascaded ANNs prediction by considering even more sources of biotic information.
